# Pattern of distant recurrence according to the molecular subtypes in Korean women with breast cancer

**DOI:** 10.1186/1477-7819-10-4

**Published:** 2012-01-08

**Authors:** Hyung Seok Park, Shinhyuk Kim, Kiho Kim, Ho Yoo, Byung Joo Chae, Ja Seong Bae, Byung Joo Song, Sang Seol Jung

**Affiliations:** 1 Department of Surgery, The Catholic University of Korea College of Medicine, Seoul, Korea

**Keywords:** breast neoplasms, neoplasm metastasis, survival, molecular subtype, estrogen, erbB-2

## Abstract

**Background:**

Distant recurrence is one of the most important risk factors in overall survival, and distant recurrence is related to a complex biologic interaction of seed and soil factors. The aim of the study was to investigate the association between the molecular subtypes and patterns of distant recurrence in patients with breast cancer.

**Methods:**

In an investigation of 313 women with breast cancer who underwent surgery from 1994 and 2000, the expressions of estrogen and progestrone receptor (ER/PR), and human epithelial receptor-2 (HER2) were evaluated. The subtypes were defined as luminal-A, luminal-HER2, HER2-enriched, and triple negative breast cancer (TNBC) according to ER, PR, and HER2 status.

**Results:**

Bone was the most common site of distant recurrence. The incidence of first distant recurrence site was significantly different among the subtypes. Brain metastasis was more frequent in the luminal-HER2 and TNBC subtypes. In subgroup analysis, overall survival in patients with distant recurrence after 24 months after surgery was significantly different among the subtypes.

**Conclusions:**

Organ-specific metastasis may depend on the molecular subtype of breast cancer. Tailored strategies against distant metastasis concerning the molecular subtypes in breast cancer may be considered.

## Background

Distant recurrence of breast cancer results in poor survival outcome and the site of the distant recurrence is also important to predict the clinical outcome [1]. The complex interaction between the primary cancer and metastatic sites includes tumor intravasation, circulation, extravasation, proliferation, and angiogenesis, and the microenvironment of the target tissue, so called "seed and soil" theory, may be associated with organ-specific metastasis in cancer patients [2,3].

The advances in the understanding of molecular subtypes analyzed by hierarchical clustering using an intrinsic gene list have identified molecular subtypes of breast cancer, and it has been noted that there is a significant difference in survival among the molecular subtypes of breast cancer [4,5]. The predictive and prognostic factors including tumor size, nodal status, lymphovascular invasion, estrogen receptor (ER) or progesterone receptor (PR), and human epithelial receptor 2 (HER2) has been investigated widely concerning molecular subtypes [6]. However, data are limited concerning differences in distant recurrence sites between the breast cancer subtypes [7]. Thus, the aim of the study was to investigate whether these subtypes were related to an organ-specific metastasis.

## Methods

### Patients

Three hundred thirteen women with primary breast cancer who underwent surgery at Seoul St. Mary's Hospital between 1994 and 2000 were enrolled in the study. Their medical data were retrospectively analyzed. Clinicopathological features including tumor size; nodal status; operation type; expression of ER, PR, and HER2; lymphovascular invasion; status of systemic therapy including endocrine or chemotherapy; radiation therapy; site and date of up to the first three recurrences; and survival data were reviewed using the medical database of Seoul St. Mary's Hospital.

### Molecular subtypes

Molecular subtypes of breast cancer were categorized according to ER, PR, and HER2 status; we defined ER or PR positive and HER2 negative as luminal-A, ER or PR positive and HER2 positive as luminal-HER2, ER and PR negative with HER2 positive as HER2-enriched, and all negative as triple negative breast cancer (TNBC) subtype.

### Distant recurrence and survival analysis

Distant recurrence was diagnosed by clinical evaluations including imaging studies or biopsy. Distant recurrence was defined as a recurrence of breast cancer developing beyond the ipsilateral or contralateral breast, chest wall, or regional lymph node including ipsilateral axillary, supraclavicular, or internal mammary lymph node. Cumulative frequency of bone (including bone marrow), lung, pleural or peritoneal, liver, brain, and other sites of metastasis (including soft tissue, pericardium, ovary, periampullary area, and other organs not elsewhere documented) during follow-up was analyzed regarding to the molecular subtypes. The site of the first distant recurrence was categorized as follows: bone, extra-bone metastasis (lung, pleural or peritoneal metastasis, liver, and other site metastasis) excluding brain metastasis, bone metastasis with synchronous extra-bone metastasis, brain metastasis, and brain metastasis with synchronous bone or extra-bone metastasis. Distant recurrence-free survival (DRFS) was defined as the time from operation to the first distant recurrence, and the cases of death without distant recurrence was censored at the time of the death. Overall survival (OS) was defined as the time from operation to death from any cause.

### ER, PR and HER2 status

ER and PR status were reviewed by medical records. Hormone receptor status was determined using an enzyme immunoassay and reported in the medical record between 1994 and 2000. The receptor status had been determined using a commercial enzyme immunoassay according to the instructions of the manufacturer (Abbott Laboratories, Chicago, IL, USA). A result exceeding 15 fmol/mg was considered positive for the presence of the particular receptor. Tissue microarray (TMA) of primary breast tissue was used for analysis of HER2 overexpression. Immunohistochemistry (IHC) or florescence in situ hybridization (FISH) for evaluating HER2 status was performed, and an IHC score of 3 positive or FISH positive was defined as positive for HER2 overexpression. The IHC method was briefly described as follows; Five-micrometer sections of paraffin-embedded tissue arrays were deparaffinized, rehydrated in a graded series of alcohol solutions and microwave-treated for 10 min in a pH 6.0 citrate buffer. The endogenous peroxidase activity was blocked using 0.3% hydrogen peroxide. The tissue arrays were processed in an automatic IHC staining machine using standard procedures (Lab Vision autostainer; Lab Vision, Fremont, CA, USA) and a ChemMate™ EnVision™ system (DAKO, Carpinteria, CA, USA). FISH was performed using the PathVysion™ HER2/CEN probe (Vysis, Downers Grove, IL, USA). The *c-erbB2 *to chromosome 17 centromere ratio was measured in at least 60 nuclei from the tumor cells, and an average score was taken. More than two copies of *c-erbB2 *for each chromosome 17 were considered to be a positive sign for *c-erbB2 *gene amplification.

### Statistics and ethics

The mean values in continuous variables are given as mean ± standard deviation. Pearson's chi-square test or Fisher's exact test were used for measuring statistical differences in categorical variables, and all statistical tests were two-sided. Kaplan-Meier method was accessed for survival analysis, and the generalized Wilcoxon test was used for estimating the difference of survival among subtypes. A Cox proportional hazard model was used for evaluating risk factors for distant recurrence-free survival and overall survival. A *P *< 0.05 was considered to be a statistically significant level. All tests were two sided. Statistical analyses were performed with SPSS 15.0 (SPSS, Chicago, IL). The present study was approved by the Institutional Review Board of the Seoul St. Mary's Hospital, the Catholic University of Korea.

## Results

Mean age of the patients was 48.7 years. Median follow-up time was 93 months (range 0-164 months). The most common subtype in the patients was luminal-A (175/313, 55.9%), followed by TNBC (62/313, 19.8%), HER2-enriched (42/313, 13.4%), and luminal-HER2 (34/313, 10.9%). Clinicopathological characteristics are shown in Table 1. All variables showed no significant difference among subtypes (*P *> 0.05).

**Table 1:** Clinicopathological characteristics according to the subtypes

	Luminal-A, n = 175(%)	Luminal-HER2, n = 34(%)	HER2-enriched, n = 41(%)	TNBC, n = 62(%)	*P*
Age					0.07
≤ 35	11(6.3)	7(20.6)	3(7.1)	6(9.7)	
> 35	164(93.7)	27(79.4)	39(92.9)	56(90.3)	
Menopausal state, n = 299					0.07
Premenopause	102	18	17	27	
Menopause	65	16	24	30	
Tumor size, n = 309					0.73
≤ 5 cm	158(90.8)	31(91.2)	35(85.4)	55(91.7)	
> 5 cm	16(9.2)	3(8.8)	6(14.6)	5(8.3)	
Nodal status, n = 306					0.59
Negative	95(54.9)	15(44.1)	25(59.5)	31(54.4)	
Positive	78(45.1)	19(55.9)	17(40.5)	26(45.6)	
Operation type					0.13
BCS	22(12.6)	1(2.9)	2(4.8)	3(4.8)	
Mastectomy	153(87.4)	33(97.1)	40(95.2)	59(95.2)	
Lymphovascular invasion					0.42
Absent	90(51.4)	17(50.0)	23(54.8)	25(40.3)	
Present	85(48.6)	17(50.0)	19(45.2)	37(59.7)	
Systemic therapy, n = 311					0.26
None	3(1.7)	1(2.9)	0	2(3.3)	
Done	172(98.3)	33(97.1)	41(100)	59(96.7)	
Radiation, n = 309					0.36
None	148(85.1)	30(90.9)	37(88.1)	56(93.3)	
Done	26(14.9)	3(9.1)	5(11.9)	4(6.7)	
Overall survival events	31(17.7)	11(32.4)	13(31.0)	15(24.2)	0.11

Distant recurrence occurred in 70 patients. Bone was the most common site of the first distant recurrence (30/70, 35.7%). Extra-bone metastasis had been shown in 25 patients, and brain metastasis was four patients. Multiple distant metastasis as the first distant recurrence was occurred in 11 patients: Eight patients with bone and extra-bone metastasis excluding brain metastasis and four patients with brain with other metastasis were presented.

The difference of the site of the first distant recurrence was significantly different among subtypes (*P *= 0.04, Table 2). While bone metastasis was the most common type of the distant recurrence in luminal-A and luminal-HER2 types (54.3% and 50.0%, respectively), extra-bone metastasis excluding brain metastasis was more frequently observed than bone metastasis in HER2-enriched and TNBC types. Brain metastasis alone developed in 16.7% of luminal-HER2 and 12.5% of TNBC types.

**Table 2:** Pattern of the first distant recurrence site

	Luminal-A(%)	Luminal-HER2(%)	HER2-enriched(%)	TNBC(%)	*P*
Bone	19(54.3)	6(50.0)	3(42.9)	2(12.5)	0.04
Extra-bone (excluding Brain)	9(25.7)	3(25.0)	4(57.1)	9(56.3)	
Bone + extra-bone (excluding Brain)	4(11.4)	1(8.3)	0	3(18.8)	
Brain	0	2(16.7)	0	2(12.5)	
Brain + other metastasis	3(8.6)	0	0	0	

Cumulative frequency data of distant metastasis site during follow-up are shown in Table 3. Brain metastasis developed in luminal-A, luminal-HER2, and TNBC types, but not in HER2-enriched type, and was statistically significant (*P *= 0.03). However, cumulative frequency of bone, lung, pleural or peritoneal, liver, and the other sites was not significantly different among subtypes (*P *> 0.05).

**Table 3:** Cumulative frequency of distant metastasis sites during follow-up

	Luminal-A(%)	Luminal-HER2(%)	HER2-enriched(%)	TNBC(%)	*P*
Bone	24(13.7)	7(20.6)	4(9.5)	6(9.7)	0.42
Lung	11(6.3)	4(11.8)	1(2.4)	7(11.3)	0.23
Pleural/peritoneal	2(1.1)	0	0	0	1.0
Liver	7(4.0)	1(2.9)	5(11.9)	5(8.1)	0.16
Brain	4(2.3)	3(8.8)	0	5(8.1)	0.03
Other	1(0.6)	0	0	2(3.2)	0.24

**Figure 1:**
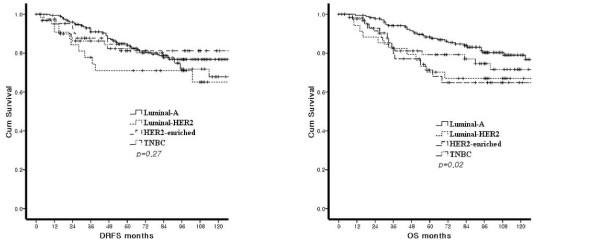
**Distant recurrence-free survival (A) and overall survival (B) according to molecular subtypes**.

**Figure 2:**
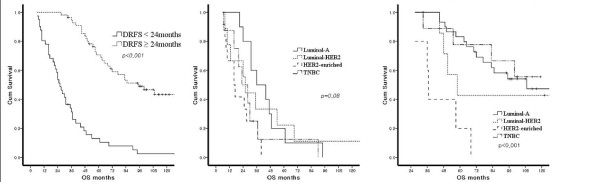
**Overall survival according to the onset of distant recurrence**. Overall survival according to the onset of distant recurrence (A) Overall survival (OS) between early and late recurrence cases (distant recurrence < 24 months and ≥ 24 months after surgery). (B) OS according to the subtypes in patients with early distant recurrence (distant recurrence within 24 months after surgery). (C) OS according to the subtypes in patients with late distant recurrence (distant recurrence ≥ 24 months after surgery).

Figure 1 illustrates DRFS and OS according to the subtypes. No significant difference in DRFS was present among the subtypes. However, OS showed differences in survival among subtypes (*P *= 0.02). When multivariate analysis for DRFS was performed (Table 4), tumor size, lymph node status, subtypes, and the site of the first recurrence were not associated with DRFS. In multivariate analysis for OS, however, HER2-enriched type, extra-bone metastasis, and brain metastasis were significantly related with poor OS (*P *< 0.05, Table 4). When the cases were stratified by onset of distant recurrence, the OS of early recurrence cases (distant recurrence within 24 months after surgery) were not significantly different by subtypes, while the OS of late recurrence cases (distant recurrence after 24 months after surgery) were significantly different by subtypes (*P *< 0.001) (Figure 2).

**Table 4:** Multivariate analysis for distant recurrence-free survival (DRFS) and overall survival (OS)

			DRFS			OS
	P	HR	95.0% CI	P	HR	95.0% CI
Subtype						
Luminal-A	Ref.			Ref.		
Luminal-HER2	0.57	1.26	0.55-2.85	0.64	1.23	0.50-2.98
HER2-enriched	0.39	1.52	0.57-4.03	0.01	3.76	1.35-10.49
TNBC	0.69	1.15	0.56-2.36	0.71	1.16	0.50-2.66
Metastatic site						
Bone	Ref.			Ref.		
Extra-bone (excluding brain)	0.67	0.87	0.46-1.64	0.05	2.05	0.99-4.23
Bone + extra-bone (excluding brain)	0.15	0.53	0.21-1.28	0.75	1.18	0.40-3.45
Brain	0.24	2.63	0.51-13.57	0.03	3.92	1.11-13.80
Brain + extra-brain	0.62	1.38	0.37-5.08	0.51	1.71	0.34-8.57
Tumor size (< 5 cm vs. ≥ 5 cm)	0.96	0.98	0.42-2.28	0.71	0.82	0.30-2.25
Nodal status (N0 vs. ≥ N1)	0.97	0.99	0.54-1.81	0.87	1.06	0.49-2.25
Age (continuous variable)	0.22	1.02	0.98-1.05	0.90	0.99	0.96-1.03

## Discussion

Since molecular subtypes of breast cancer have been identified [4], many studies have explored the clinical value of the molecular subtypes of breast cancer [5,8,9]. However, most studies have focused on clinicopathological features, different risks of recurrence, and response to systemic therapies according to the molecular subtypes [9-11], and only a few studies have described different distant metastatic patterns according to molecular subtypes [7,12]. A retrospective study analyzed a large dataset and demonstrated a distinct pattern of metastatic behavior of the molecular subtypes using a validated six-marker immunohistochemical panel applied to tissue microarrays, even though the clinicopathological features of the molecular subtypes that might influence the pattern of distant recurrence differed among the molecular subtypes [7]. The current study demonstrated that different patterns of distant recurrence among the subtypes with well-balanced clinicopathologic features remained in terms of the first distant recurrence site, frequency of brain metastasis, overall survival, and onset of distant recurrence.

Distant recurrence-free survival was not different among the subtypes, yet OS showed significant differences. Generally, patients with bone metastasis show better survival than those with visceral metastasis [13], and bone metastasis is more common in luminal types than the other subtypes [7,12]. Therefore, the discordance between DRFS and OS might derive from the difference of the sites of the distant recurrence of the four subtypes.

Previous studies have reported a significant difference in the onset of recurrence according to hormone receptor status and hormonal therapy [14-17]; usually low-ER positive or ER negative tumors are associated with early recurrence [14,17]. In the current study, early distant recurrence was not related to the molecular subtypes. This was not concordant with the previous studies that demonstrated TNBC- or ER-negative tumors are related with early relapse [7,14,17]. However, interpretation of this discordance needs to be done cautiously, because the definition of early relapse and study design of the previous studies are so heterogenous that directly comparison with each study cannot be made. The patients with HER2 overexpression, luminal-HER2 and HER2-enriched subtypes, with late distant recurrence were associated with poorer survival than luminal-A and TNBC; these findings may be associated with lack of trastzumab therapy because this therapy was approved and covered by the government and national insurance system in Korea in the mid-2000s. In addition, race may influence the discordance because disparities in breast cancer prognostic factors by race may exist [18]. Regarding the study population of the current study, the ethnicity is also considered to interpret the discordance from previous studies which were mainly from Western countries [7,14]. A previous Taiwanese study mentioned that TNBC in Taiwan may have different prognostic factors and features compared with Western countries, possibly due to genetic heterogeneity [19]. In addition, a previous study reported gene expression profile differences in breast cancer between African American and non-African American women supporting that genetic heterogeneity in the tumor microenvironment from different ethnicity may account for the difference in the site of distant metastasis [20]. There might be an ethnic factor related to metastatic patterns of breast cancer, and further investigations are needed.

The pattern of distant recurrence in TNBC has been well-investigated because of its distinct pattern [21-24]. Brain metastasis is common in TNBC; previous studies have reported an incidence of brain metastasis of 6%-10% [7,12,23,24], similar to our results. These previous studies also reported about an incidence of CNB metastasis of about 10% among patients with early stage HER2 positive tumor, which is concordant with the 8.8% of brain metastasis in luminal-HER2 type, but higher than the rate found for the HER2-enriched type. The incidence of brain metastasis in patients with HER2-positive metastatic tumor who received trastuzumab has been reported to be 25%-34% [25,26]. The frequency of brain metastasis in patients with HER2-positive metastatic tumor, luminal-HER2 and HER2-enriched metastatic tumor, in the current study (3/19, 15.7%) is lower, and is perhaps associated with the lack of trastuzumab therapy. In the absence of trastuzumab therapy for HER2-positive breast cancer patients, other visceral metastases may develop and earlier failure in other visceral organ may cause death before the development of brain metastasis.

The limitations of the study are the small number of the cases, its retrospective design, and the fact that systemic therapy guidelines during the era of this cohort are not representative of current practice guidelines. A major strength of the study is that the well-balanced clinicopathological features in this cohort allowed more clear demonstration of the metastatic pattern according to molecular subtypes. In addition, a full review of the medical records permitted a check of the detailed documented metastatic sites and date of up to the first three distant recurrences, providing a more apparent pattern of cumulative frequency of distant metastasis sites as well as onset of distant metastasis.

## Conclusions

In conclusion, this study demonstrates that the pattern of distant metastasis is different among the molecular subtypes as defined by ER, PR, and HER2 status, and may contribute to an understanding of molecular subtype that will allow for tailored therapy for metastatic breast cancer.

## Competing interests

The authors declare that they have no competing interests.

## Authors' contributions

HSP contributed mainly in the design, literature review and writing of the article. Collection and assembly of the data was performed by BJC and HSP. BJS provided the idea, planned, edited and approved the written work. SK, KK, HY, BJC, JSB, and SSJ gave valuable advices and edited the discussion. Both BJS and SSJ also provided administrative supports. All authors read and approved the manuscript.
